# Novel report of an osteogenic tumor in a late Jurassic Mamenchisaurid from Thailand

**DOI:** 10.1111/joa.14266

**Published:** 2025-04-24

**Authors:** Siripat Kaikaew, Suravech Suteethorn, Anusuya Chinsamy

**Affiliations:** ^1^ Department of Biological Sciences University of Cape Town Cape Town South Africa; ^2^ Department of Biology, Faculty of Science Mahasarakham University Maha Sarakham Thailand; ^3^ Palaeontological Research and Education Centre Mahasarakham University Maha Sarakham Thailand; ^4^ Dinosaur Research Unit Mahasarakham University Maha Sarakham Thailand

**Keywords:** bone tumor, Eusauropod, lesion, neoplasm, periosteal reactive

## Abstract

Here we report on an osseous abnormality and multiple fractures in an ulna of a subadult basal Eusauropod (Mamenchisauridae) from the Late Jurassic Phu Kradueng Formation in Thailand. The anatomical deformities were studied using a multi‐method approach that included an assessment of its gross morphology, computed tomography (CT), and osteohistology to aid in its diagnosis. The intracortical lesion in the bone is irregularly shaped, has well‐defined margins with scattered irregular bony trabeculae especially in its center, and it is surrounded by sclerotic bone and spiculated periosteal reactive tissue. The analysis of the radiology and the histopathological characteristics indicates that the lesion in the ulna is an osteogenic tumor, although we are unable to confidently commit to a more specific diagnosis. CT scan data indicated that the multiple fractures evident in the ulna occurred postmortem and are unrelated to the pathology. This is the first report of an osteogenic tumor in a basal Eusauropoda.

## Graphical abstract

Here we describe a pathology in an ulna of a Late Jurassic mamenchisaurid from Thailand. We use anatomical descriptions, CT scanning, and bone histology to identify the pathology as an osteogenic tumor.

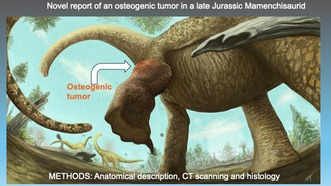

## 1 INTRODUCTION

Mamenchisauridae are the dominant early diverging Eusauropod clade in the Middle and Late Jurassic of East Asia, including Thailand. These dinosaurs have the distinction of being the sauropods with the longest necks (Mannion et al., 2019; Moore et al., 2020, 2023; Ouyang & Ye, 2002; Ren et al., 2020; Russell & Zheng, 1993; Suteethorn et al., 2012; Tang et al., 2001; Upchurch et al., 2004; Wu et al., 2013; Xing et al., 2015; Young, 1958; Zhang et al., 1998). The mamenchisaurid record in Thailand derives from many locations, including the world‐renowned Upper Jurassic Phu Noi locality in the lower part of the Phu Kradung Formation (Boonchai et al., 2020; Buffetaut et al., 2014; Tong et al., 2015). Among the large number of mamenchisaurid bones recovered from Phu Noi, an anomalous osseous growth was observed in an ulna, which was inferred to be a pathologic feature.

Paleopathology is the study of the evidence of injury and disease that inflicted prehistoric animals (e.g., Samathi et al., 2023; Woolley et al., 2021). Paleopathological diagnoses provide key information on the macroevolutionary origin of disease as well as behavioral and physiological inferences of extinct organisms (e.g., Chinzorig et al., 2023). Early paleontological studies of pathologies were investigated primarily through gross morphological observations, which are of limited diagnostic utility. More recently, a combined approach using multiple analytic methods, including advances in bone microstructural analyses using plain/polarized light microscopy, as well as computed tomography has been shown to be invaluable for paleopathological research (e.g., Straight et al., 2009). This is particularly true for the diagnosis of disease in prehistoric animals (David & Zimmerman, 2010; Hedrick et al., 2016), as well as for understanding the response of bone at both the macroscale and the microscale (histological level) (Anné et al., 2016; Chinzorig et al., 2023; Hedrick et al., 2016; Woolley et al., 2021).

Neoplasms (bone tumors) are defined as a mass of localized tissue growth that proliferates at an abnormally high rate as a result of defects in the regulation of cell division (Odes et al., 2016; Randolph‐Quinney et al., 2016; Surmik et al., 2023). A tumor may be benign or malignant in nature; malignant tumors are often colloquially referred to as cancer (Odes et al., 2016). Several neoplasm studies have been conducted on extinct vertebrates, and they include osteosarcoma, osteoblastoma, osteoid osteoma, Ewing's sarcoma, osteochondroma, etc. (Hamm et al., 2020; Rothschild et al., 2023). More specifically, among the extinct vertebrate bone tumors have been reasonably well documented: For example, osteosarcoma in the turtle *Pappochelys rosinae* (Haridy et al., 2019); Ewing's sarcoma, which exhibited spiculated periosteal reactive bone tissue (i.e., hair‐on‐end tissue) in a basal sauropod *Isanosaurus attavipachi* (Jentgen‐Ceschino et al., 2020); an osteoblastic tumor in the titanosaurid *Bonitasaura salgadoi* (Gonzalez et al., 2017); an osteosarcoma in the ceratopsian *Centrosaurus apertus* (Ekhtiari et al., 2020); an ameloblastoma in the hadrosaurid *Telmatosaurus transsylvanicus* (Dumbravă et al., 2016); langerhans cell histiocytosis in an undetermined hadrosaur (Rothschild et al., 2020); osteochondroma in some tyrannosaurids; and indeterminate tumors in the non‐avian theropods *Allosaurus* and *Dilophosaurus* (Baiano et al., 2024), as well as an osteoma in the mosasaur *Platecarpus* (de Souza Barbosa et al., 2016).

The current study investigates the pathologic Thai mamenchisaurid ulna (PN14‐108) that was discovered by the Palaeontological Research and Education Centre team in 2014. Here, we apply complementary gross morphological observations, CT scanning, and osteohistological information to describe the pathology and to apply a differential diagnosis. We also utilize the osteohistology data to assess the ontogenetic status of the dinosaur. Overall, this study provides a better understanding of the biology of the Eusauropoda and the diseases that afflicted them.

## 2 MATERIALS AND METHODS

### 2.1 Ulna, specimen PN14‐108

**FIGURE 1:**
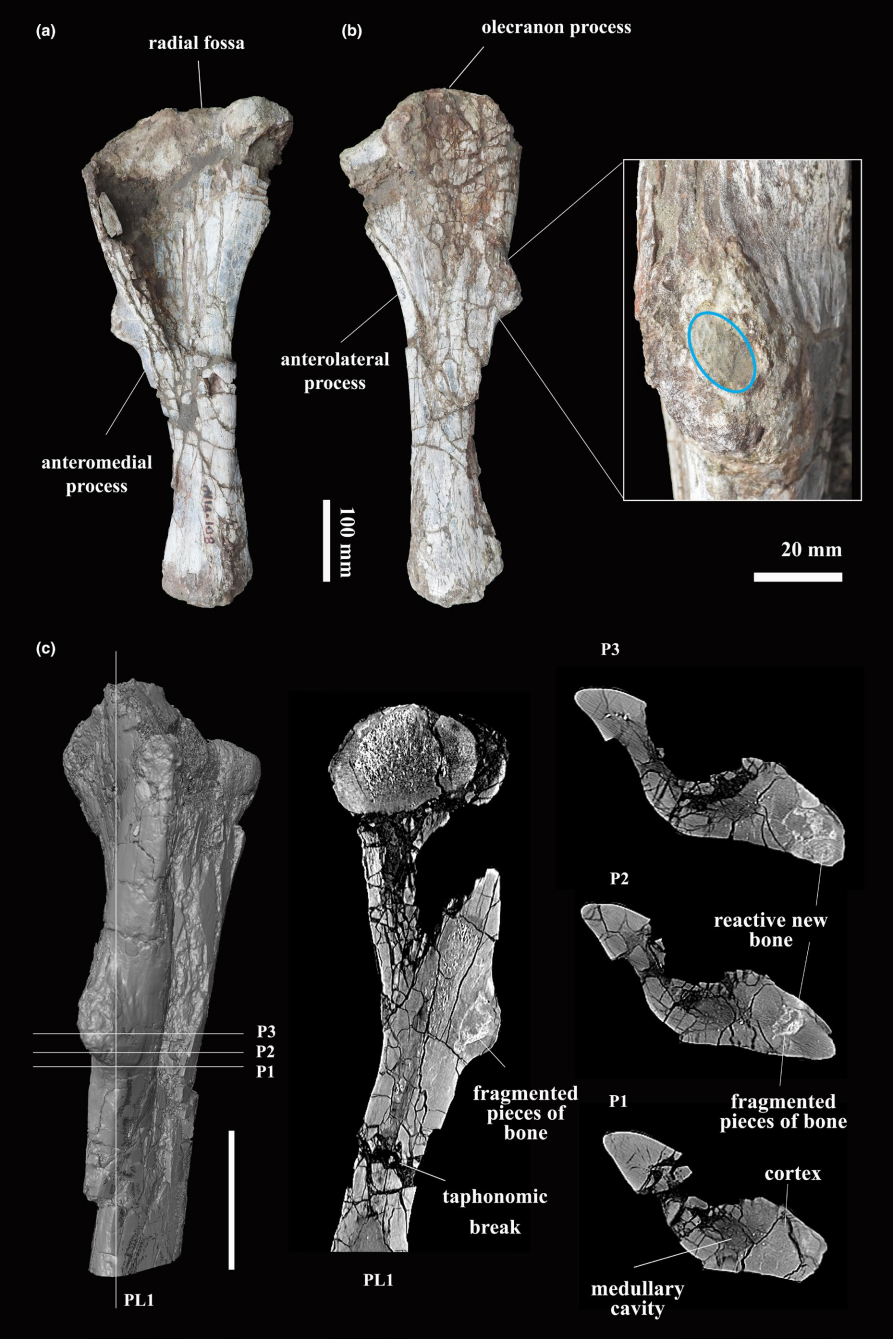
Mamenchisaurid ulna PN14‐108. (a) Gross external morphology of the ulna in lateral view. (b) Gross external morphology of the ulna in medial view. The box shows close‐up images of the external area that connects intracortically. (c) A 3D reconstruction in posterior view based on CT data and virtual thin sections showing the pathologic bone. PL1 indicates a sagittal view, whereas P1–P3 indicates cross‐sectional views. Scale bar equals 100 mm.

In 2008, a combined team comprising members of the Sirindhorn Museum, Department of Mineral Resources (DMR) and French paleontologists undertook excavations at the Phu Noi locality in the Kalasin province of Northeastern Thailand. The Phu Noi locality is located on the flank of a small hill near the village Dinji, Kham Muang District, Kalasin Province. The locality is in the Phu Kradung Formation and yields the brown, gray, and greenish mudstones typical of the Formation (Boonchai et al., 2020; Buffetaut et al., 2014; Tong et al., 2015). The age of the Phu Kradung Formation is mainly dated to the Late Jurassic but includes some early Cretaceous deposits (Buffetaut et al., 2014; Racey & Goodall, 2009; Tong et al., 2015). This study focuses on the mamenchisaurid ulna, PN14‐108 (Figure 1). The specimen is incomplete in that it is missing the proximal surface of the medial side. It has been encased with epoxy to stabilize the poorly preserved regions (Kaikaew, 2022; Suteethorn et al., 2012). The posterior process of the ulna (PN18‐104) is weakly developed, and the olecranon process is not prominent, resembling other basal sauropods. The ulna has a distinctive triradiate proximal surface as seen in *Chuanjiesaurus anaensis*, and the midshaft is transformed into an ellipse‐shaped cross‐section as seen in mamenchisauridae: *C. anaensis*, *Huangshanlong anhuiensis*, *Anhuilong diboensis*, and titanosaur *Rapetosaurus kiausei* (Ren et al., 2020, 2021; Upchurch et al., 2021).

### 2.2 Institutional abbreviations

DMR, Sirindhorn Museum, Department of Mineral Resources, Thailand; PN, Phu Noi locality, Kalasin province, Thailand; PRC, Palaeontological Research and Education Centre, Mahasarakham University, Thailand.

### 2.3 CT scanning

Specimen PN14‐108 was scanned with a TOSHIBA Aquillion 64 scanner (Faculty of Medicine; Mahasarakham University, Thailand) with an acceleration voltage of 120 kV and a current of 300 mA. The thickness of the slices was 0.4 mm. Raw data from the scan were reconstructed using a high‐spatial‐frequency (bone) algorithm, producing 512 slices. ImageJ and 3D slicer (version 4.11.0) software was used for the three‐dimensional reconstruction and segmentation of the specimen. In the CT image, three cross‐sections (P1–P3) and one sagittal section (PL1) were taken in the region of the metaphysis that includes the pathology (Figure 1c).

### 2.4 Histological preparation and analysis

**FIGURE 2:**
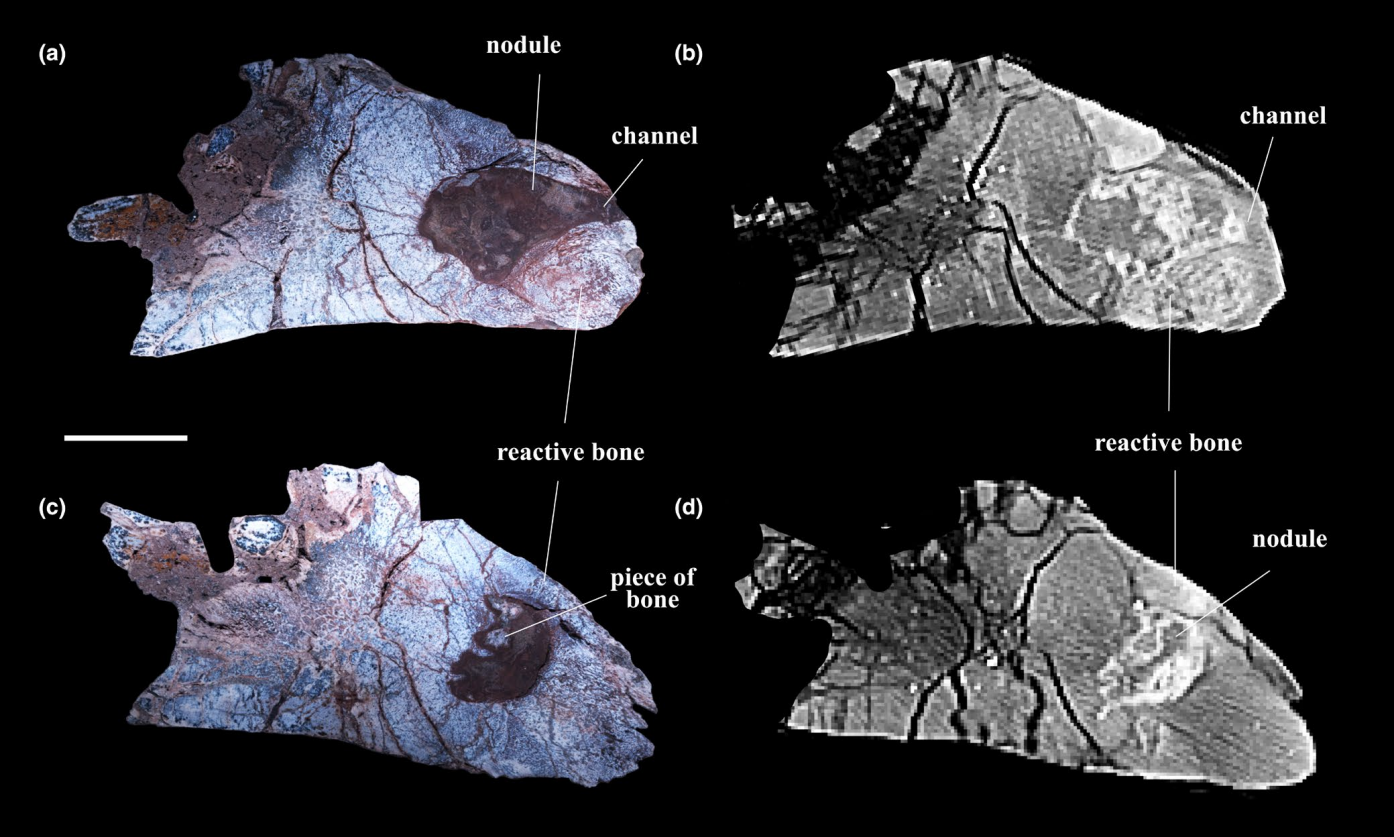
(a) and (c) Cross‐sections of the respective pathological regions, P3 and P2 (as indicated in Figure 1c); (b) and (d), the CT scan images of the pathological regions on the posterior side of PN14‐108. Scale bar equals 20 mm.

Histological thin sections of PN14‐108 were performed in the Palaeontological Research and Education Centre in Thailand, and at the Department of Biological Sciences at the University of Cape Town, using standard paleohistological techniques (Chinsamy & Raath, 1992; Padian & Lamm, 2013). The ulna was cut transversely through the lesion at the posterior metaphysis (Figure 1c, P1–P3). Bones were sectioned as indicated in Figures 1 and 2. High‐quality photomicrographs were taken using normal and polarized light using a Nikon Eclipse E200 polarizing microscope and a Zeiss AX10. These photographs were processed using the NIS‐Elements software and Kolor Autopano Giga (APG). The histology terminology used in this manuscript follows Chinsamy‐Turan (2005) and Francillon‐Vieillot et al. (1990).

## 3 RESULTS

### 3.1 Osteopathological analysis

The ulna (PN14‐108) of the mamenchisaurid from Phu Noi is generally well preserved. The bone is undeformed, but the proximal head of the medial side has some breakage. The bone surface appears to be damaged because of postdepositional taphonomic processes (Figure 1). The ulna clearly shows a distinctive pathology in the metaphyseal region, although the proximal, midshaft, and distal articular surfaces are unaffected. The pathology presents as a bony expansion on the metaphyseal aspect of the ulna, which is elliptical in shape in posterior view (Figure 1). The dimensions of the pathologic area are approximately 80 mm in proximo‐distal length and 35.9 mm in latero‐medial width (Table 1). The surface of the lesion varies from roughly smooth (proximally) to irregular (distally). In lateral and medial views, the outgrowth protrudes to about 46 mm from the normal surface of the shaft (Figure 1). On the surface of the bone, a shallow sediment‐filled pit is visible (Figure 1b).

**TABLE 1:** Anatomical measurements of the ulna PN14‐108 and the associated pathologic region.

Measurements	(mm)
Total length	606.23
Total width of the metaphysis shaft	161.75
Total width of the diaphysis shaft	93.16
Length of the pathology	80.01
Width of the pathology	35.9
Height of the pathology	46.08
Length of the lesion nodule (CT data)	41.59
Width of the nodule (CT data)	16.04
Height of the nodule (CT data)	20.56
Width of the channel (CT data)	2.65

In the CT images, it is apparent that some taphonomic modifications are present. The metaphyseal cortex has an abundant number of fractures, and the medullary cavity is compressed to a narrow area in the CT image (Figure 1c). In the diaphysis, a large break as well as multiple small bone fragments occur in sediment‐filled cavities. There is also no reactive bone (i.e., fracture callus) formation in the anterior–posterior cortices (in the sagittal plane) (Figure 1c). The cavities are primarily sites of calcite precipitation and are infilled with a mud‐matrix, which provides taphonomic evidence that the large breakages in these areas were associated with postmortem crushing and burial (Kato et al., 2020; Peterson & Vittore, 2012). The original cortical bone is readily distinguishable from the pathologic bone tissue. An enlarged osteolytic region is visible, and it measures 34.7 mm in length and 16.5 mm in width (Figures 1 and 2). In the cross‐section of the posterior side, the osteolytic area is well defined by a hyperdense rim which is infilled by sediments. Thus, giving it a nodule‐like appearance (Figures 1c and 2). The osteolytic area is irregularly shaped, and in some areas, the lesion extends right up to the periosteal surface (Figure 2a). At its center is a large lytic area which has areas of mineralization and fragmented bones. This nodule is surrounded by a hyperdense area which is the sclerotic cortical bone (Figures 1 and 2) (Jentgen‐Ceschino et al., 2020; Villa et al., 2019).

### 3.2 Paleohistological analysis

**FIGURE 3:**
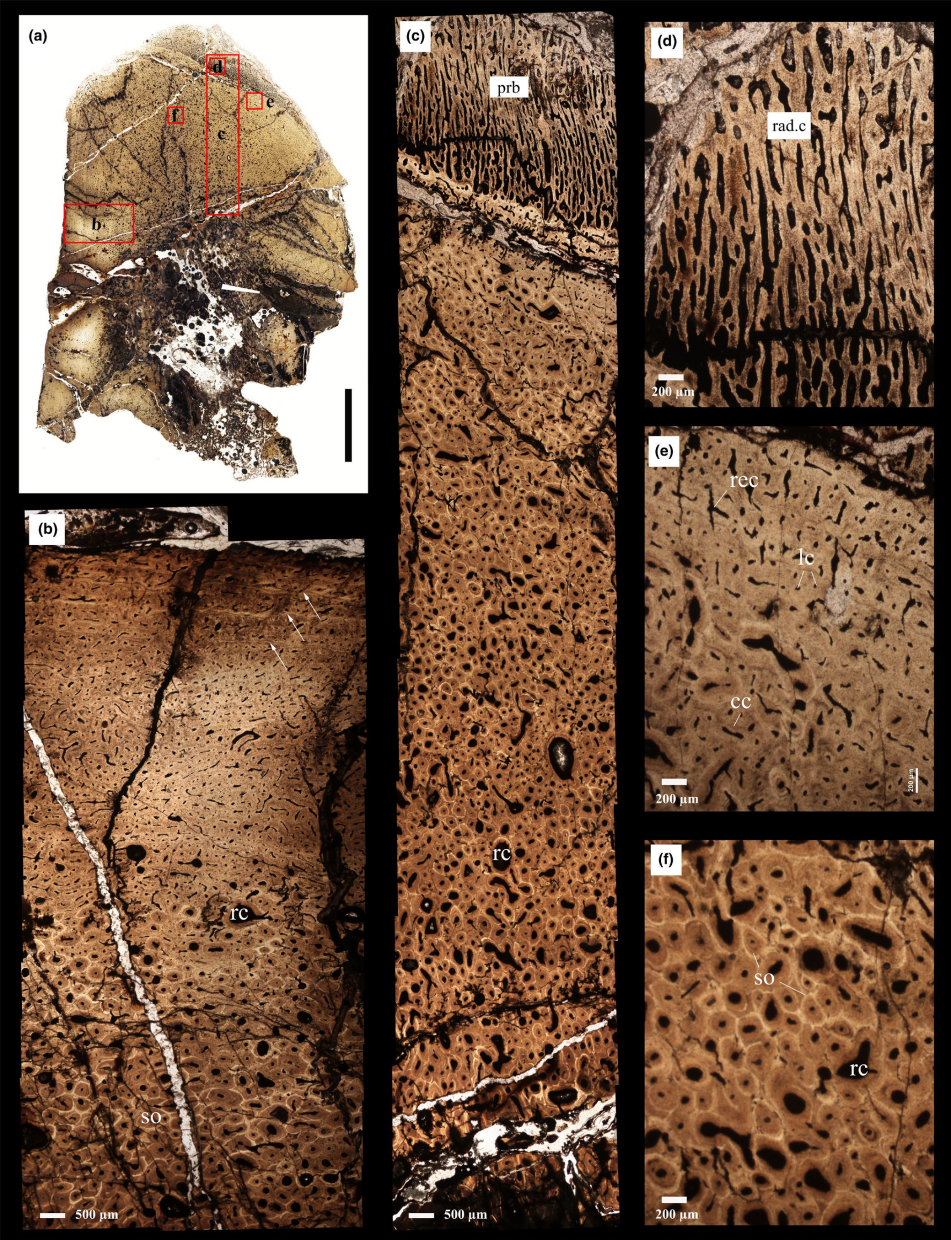
Osteohistology of the P1 cross‐section of PN14‐108 (as indicated in Figure 1c). (a) Overall low magnification view of the cross‐section; red frames indicate the areas that are magnified in other images. (b) The lateral side of the posterior process showing intensive secondary reconstruction in the deeper parts of the compacta with many secondary osteons (so) and resorption cavities (rc). This region is overlain by a region comprising predominantly of primary bone, that is, fibrolamellar tissue with abundant longitudinal, circular, and reticular oriented vascular canals. Toward the subperiosteal surface, three growth marks (white arrows) interrupt the deposition of the fibrolamellar bone tissue. (c) The general view of the transition between pathological bone tissue (uppermost region) and non‐pathologic (lower region). The outermost cortex comprises of periosteal reactive bone (prb) with abundant radially organized bony spicules. rc and arrow indicates resorption cavities. (d) Higher magnification of the prb showing details of the hair‐on‐end spiculae (i.e., bony spicules perpendicular to the periosteal surface) and the large number of radially organized vascular canals (rad.c). (e) Higher magnification of the tissue underlying the prb showing longitudinally (lc), circumferentially (cc), and reticularly (rec) oriented primary osteons. (f) Secondary reconstruction in the inner cortex with several generations of secondary osteons, as well as many resorption cavities (rc).

The transverse thin section P1 of PN14‐108 (Figure 1c) appears to have a homogenous compact texture from the inner region to the middle cortex of the bone (i.e., about two thirds of the cortex) (Figure 3a,b). Primary osteons are visible toward the outer parts of the cortex, whereas multiple generations of secondary osteons are abundantly visible in the deeper parts of the compacta, although the tissue never reaches dense Haversian bone levels (Figure 3b,f). Numerous enlarged resorption cavities without any secondary deposits of lamellar bone tissue are visible in the deeper parts of the compacta (Figure 3e). In the normal primary bone part of the compacta, fibrolamellar bone is the predominant tissue type with a high degree of small longitudinal, circular, and reticular oriented vascular canals (Figure 3b). This area differs from the outer cortex of the medial and lateral sides of the bone, which comprises fibrolamellar bone predominantly with plexiform vascularization. Additionally, at least three growth marks are visible in the outer cortex, although an external fundamental system (EFS) indicating skeletal maturity is not present (Figure 3b). In the pathologic area (Figure 3c,d), the outermost cortex is dominated by periosteal reactive bone with abundant radially organized spicules of bone and radially arranged vascular canals (e.g., Chinsamy & Tumarkin‐Deratzian, 2009). The bony spicules are perpendicular to the surface of the bone and can be described as “hair‐on‐end” periosteal reactive bone tissue (e.g., Jentgen‐Ceschino et al., 2020; Redelstorff et al., 2015). The radial canals in this region are substantially larger than the rest of the cortex. This region exhibits a high density of large spherical osteocyte lacunae (Cerda et al., 2014), which appear to be more densely distributed in the lower parts of the spiculae of bony struts.

**FIGURE 4:**
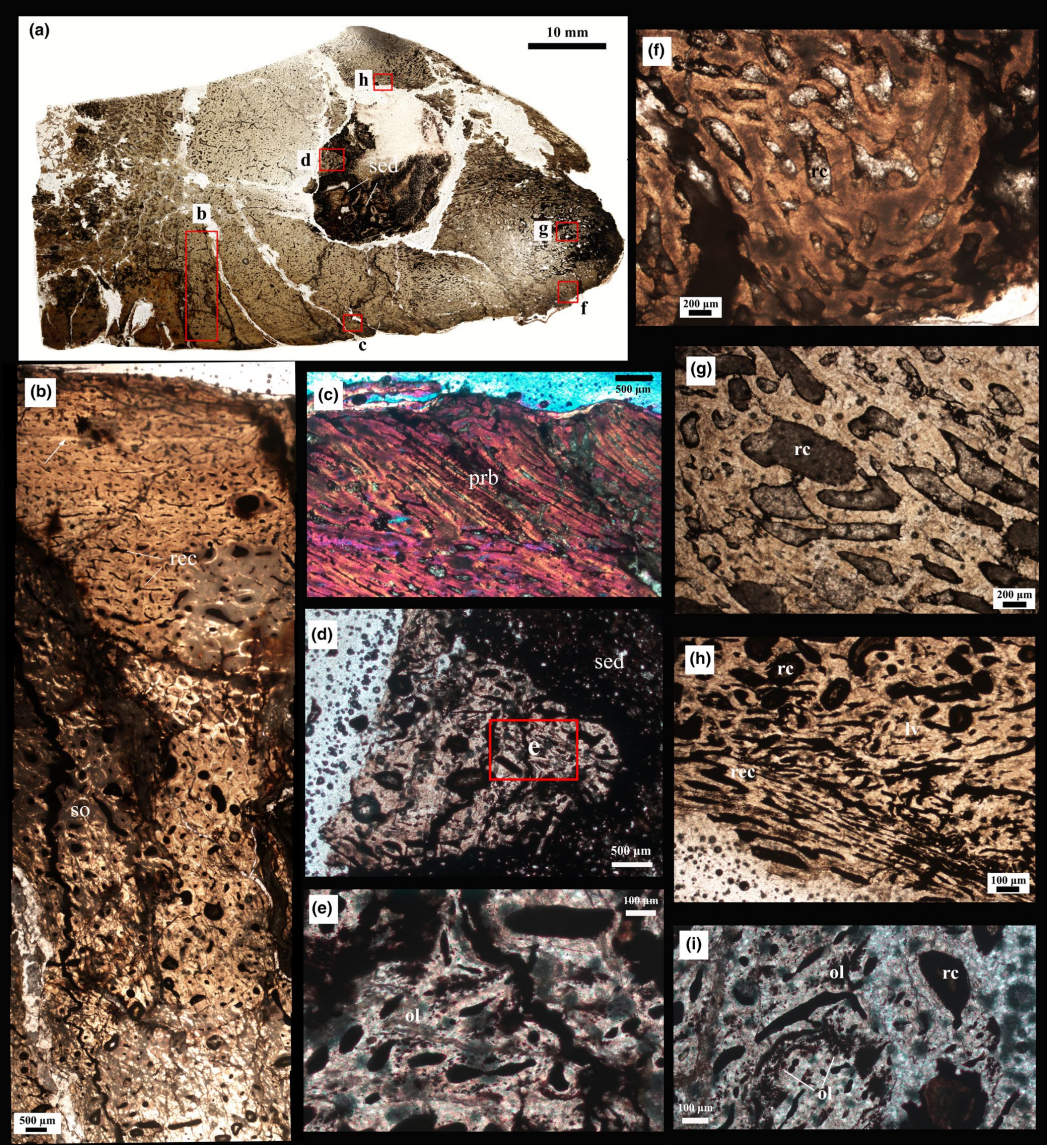
Osteohistology of the P2 cross‐section of PN14‐108 (as indicated in Figure 1c). (a) A low magnification view of the overall cross‐section; red frames indicate the magnified images illustrated in (b), (c), (d), (f), (g), and (h). (b) Osteohistology of the medial side of the posterior process of PN14‐108. In the deep parts of the cortex, there are many secondary osteons (so) and several resorption cavities. The arrow indicates a growth mark. Reticular oriented vascular canals (rec) are also indicated. (c) Oblique, radially organized vascular canals of the spiculated periosteal reactive bone tissue (prb) are visible in the outermost part of the cortex. (d) General view of a piece of bone located within the sediment (sed). The red frame indicates the region magnified in (e). (e) High magnification view of the framed region in (d) showing details of the bone tissue. Dense patches of osteocyte lacunae (ol) are visible. (f) and (g) Reactive new bone comprised of woven bone tissue and resorption cavities (rc) of varying sizes. (h) A low magnification view of the reactive new bone showing the various sizes of resorption cavities (rc), reticular canals (rec), and longitudinal vascular canals (lv) with rounded osteocyte lacunae. (i) High magnification view of reactive new bone showing dense patches of osteocyte lacunae (ol) and enlarged resorption cavities (rc).

**FIGURE 5:**
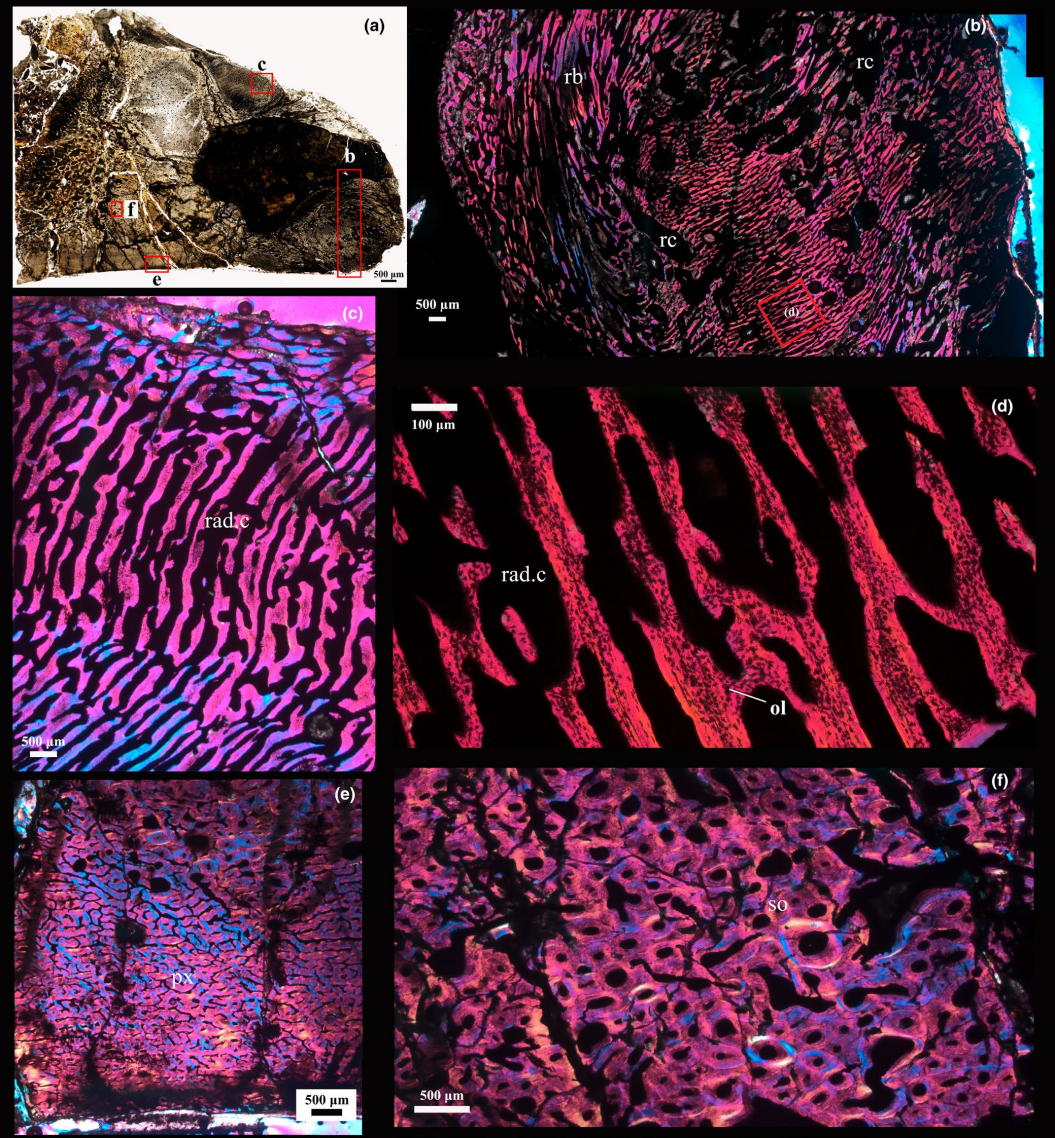
Osteohistology of the P3 cross‐section of PN14‐108 (as indicated in Figure 1c). (a) A low magnification view of the overall cross‐section; red frames indicate the magnified images illustrated in (b), (c), (e), and (f). (b) Overview of the region showing the osteohistology of the posterior process of PN14‐108 showing reactive new bone (rb) and many enlarged resorption cavities (rc). The outer cortex is dominated by reactive bone (rb) with abundant radially organized spicules and various sizes of resorption cavities (rc). The red frame indicates the region magnified in (d). (c) Showing the oblique hair‐on‐end spiculae in the outermost cortex with many radially organized vascular canals (rad. c.). (d) High magnification view of the reactive new bone (as indicated in the red box frame in b) showing the large radially organized vascular canals (rad. c.) with many rounded osteocyte lacunae (ol). (e) Detail of the outer cortex at the lateral side with vascular canals arranged in a plexiform pattern (px) and several resorption cavities. (f) The outer cortex along the lateral side is dominated by secondary osteons (so) and many enlarged resorption cavities.

In the transverse thin sections P2 and P3 (Figures 4 and 5), the normal area of the cortical bone makes up about a third of the inner bone wall, which is composed of fibrolamellar bone tissue with predominantly longitudinal and reticular organized vascular canals, whereas the medial side exhibits a plexiform vascularization as well as small longitudinal canals. About two thirds of the inner cortex comprises multiple generations of secondary osteons, consistent with extensive secondary reconstruction (Figures 4b and 5e,f). The outermost cortex of the pathologic region is overlain by radially organized fibrolamellar bone tissue that is slightly oblique to the original periosteal surface (Figures 4a,c and 5b–d). This area exhibits an abundance of plump osteocyte lacunae (Figure 4d). A large nodule with an undulating scalloped edge occurs within this section (Figure 4a). The nodule not only appears to be infilled with sediment (quartz, sand, and mud), but also contains pieces of bone near its margin (Figure 4a,d,e). In addition, overlying the nodule is a thick irregular region of bone tissue which has many erosion cavities of varying sizes and many longitudinal and reticularly organized vascular canals (Figures 4f–h and 5b–d). The osteocyte lacunae are round, different sized, and densely distributed as compared to the other areas (Figures 4e,i and 5d). These features suggest that this tissue represents pathologic reactive new bone formation (e.g., Jentgen‐Ceschino et al., 2020). Moreover, in the outermost part of thin section P3, a distinct channel is visible that extends intracortically and opens to the external margin of the bone (Figures 1, 2a and 5a).

## 4 DISCUSSION

Here we use the anatomical, osteohistological, and CT scan data to attempt to diagnose the pathology evident in the ulna of the mamenchisaurid. In addition, we also utilize the histological data to assess the ontogenetic status of the mamenchisaurid, and using a combination of the CT scan data and histological information, we propose that some breakages in the bone appear taphonomic and unrelated to the pathology.

### 4.1 Differential diagnosis of the lesion in the ulna, PN14‐108

In our analyses, the CT images in conjunction with the thin sections of the bone provide complementary information regarding the pathologic processes that affected the ulna (PN14‐108) of the Thai mamenchisaurid. Gross examination of the specimen revealed a distinctive, abnormal bulge on the posterior side of the metaphysis of the bone shaft. The bone growth and expansion on the metaphysis, as well as the uneven surface textures, are similar to several bone pathologies previously described, including osteomyelitis of the right tibiae (MOR 005‐T42) of the hadrosaurid *Maiasaura peeblesorum* (Cubo et al., 2015), post trauma of the caudal vertebra (MPCA‐Pv SM2/19) of the hadrosaurid *Bonapartesaurus rionegrensis* (Cruzado‐Caballero et al., 2021), acute periostitis of a radius (NHCC LB396) of an undetermined gorgonopsid (Kato et al., 2020), and osteomyelitis of a left fibula (FMNH PR2081) of the non‐avian theropod *Tyrannosaurus rex* (Hamm et al., 2020). Additionally, this lesion also resembles neoplasms seen in the osteosarcoma described in a fibula (TMP 2014.050.0192) of the ceratopsian *C*. *apertus* (Ekhtiari et al., 2020) and ameloblastoma of the dentary of the hadrosauroid *T. transsylvanicus* (Dumbravă et al., 2016). Thus, given that the lesion is superficially like several different types of pathologies, we applied differential diagnoses to narrow down what it might be.

The CT images and histological section PN14‐108 show a nodule‐like structure located within the cortical bone. The images reveal the enlarged localized destruction with well‐defined borders and are surrounded by a less sclerotic reactive growth. This nodule is infilled with sediment and shows fragments of bone (with irregular bony trabeculae). The surface texture of the lesion is irregular in shape and is porous. Moreover, histologically, the lesion shows reactive new bone formed by woven bone and is richly vascularized. A distinctive hair‐on‐end periosteal reactive growth is visible at the lateral side. Thus, the lesion affecting the ulna from Phu Noi locality is characterized by both aggressive (periosteal reactive growth) and nonaggressive radiological (well‐defined borders of the lesion) features, as well as other distinctive histological features. Considering clinical publications, as well as the veterinary pathology and paleopathological literature, it appears that the pathologies that could cause a solitary osteolytic lesion within the cortex of a bone could have resulted from osteomyelitis, an intraosseous abscess, and bone tumors such as chondroblastoma, osteoid osteoma, osteoblastoma, intracortical osteosarcoma, and nonneoplastic (e.g., Buikstra, 2019; Jennin et al., 2011; Maxie, 2015; Olson & Carlson, 2017; Rothschild et al., 2023; Surmik et al., 2023; Thompson & Dittmer, 2017) (Table S1).

Based on the location of the lesion in our mamenchisaurid ulna, the osteolysis in the intracortical metaphysis is inconsistent with the osteolytic location of a chondroblastoma (Buikstra, 2019; Jennin et al., 2011; Rothschild et al., 2023; Surmik et al., 2023). The internal nodule of PN14‐108 exhibits a mixed lytic and sclerotic region, but its lytic density is dissimilar to that observed in abscesses and intracortical osteosarcomas (e.g., Griffith et al., 1998; Menendez et al., 2022; Rothschild et al., 2023). Although many of the characteristics of osteomyelitis are similar to those of neoplasms (e.g., Olson & Carlson, 2017) the lesion in PN14‐108 has well‐defined margins and does not have the sequestra typical of chronic osteomyelitis (Rothschild et al., 2023; Surmik et al., 2023); the channel that appears to connect intracortically seems to be taphonomic. The spiculated hair‐on‐end reactive growth perpendicular to the bone surface has been described in many vertebrates, such as the sauropodomorph *Isanosaurus* (Jentgen‐Ceschino et al., 2020), the titanosuchid *Jonkeria parva* (Shelton et al., 2017), and the thyreophoran *Stegosaurus* (Redelstorff et al., 2015). Such spiculated bone is indicative of aggressive periosteal reactive growth in malignant tumors or in severe infections and is a consequence of fast bone deposition in a short period of time (Alencar et al., 2020; Jentgen‐Ceschino et al., 2020; Rana et al., 2009). Since nonneoplastic hemangioma, amyloidosis, sarcoidosis, and chondromyxoid fibroma do not present with periosteal reactive bone tissues, we exclude them as possible diagnoses.

Osteoid osteoma and osteoblastoma have similar histological features as described for our mamenchisaurid ulna but differ in terms of size and location as well as other radiographic features. Lesions caused by osteoblastomas are often greater than 2 cm in size, with well‐defined margins, and are radiolucent with scattered areas of mineralization that have the density of trabecular bone, especially in the center, and are often surrounded by a zone of sclerotic reactive bone (Atesok et al., 2011; Buikstra, 2019; Galgano et al., 2016; Olson & Carlson, 2017; Rothschild et al., 2023). Given that the osteolysis of PN14‐108 is large, irregularly shaped without a central nidus, and shows bony destruction and scattered mineralization, we do not consider the lesion of PN14‐108 to be the result of an osteoid osteoma, which typically has an oval or round lucent lesion with a densely sclerotic center and is surrounded by a thick, reactive rim of sclerosis (e.g., Barlow et al., 2013; Kutlu et al., 2017; Olson & Carlson, 2017; Rana et al., 2009). Could the pathology evident in the ulna of our mamenchisaurid be an osteoblastoma? Aside from humans, osteoblastomas are seldom reported in the veterinary literature (Olson & Carlson, 2017), but they have been described in the distal humerus of a cat (Kirk et al., 2019), the distal metaphysis of a pony (Goedegebuure et al., 1983), a vertebra of *Australopithecus sediba* (Randolph‐Quinney et al., 2016), a vertebra of an unnamed sauropod (Stokes et al., 2016), and a fragment of a carapace of a Miocene turtle, *Syllomus aegyptiacus* (Rothschild et al., 2012). Usually, osteoblastomas have an intraosseous expansile mass with minimal to no periosteal reactive bone deposition. The expansile mass is usually composed of a soft tissue spindle cell cancer that causes lysis of the existing medullary or cortical bone and enlargement of the pathologic area. Thus, osteoblastomas should appear hypodense on CT due to the loss of bone and replacement with a soft tissue cancer. In our mamenchisaurid ulna, there does not appear to be a distinctive osteolytic region. The presence of the nodule could perhaps have been the lysis cavity infilled by sediment during fossilization, but we cannot be certain that this is indeed the case. Furthermore, contrary to osteoblastomas, the mamenchisaurid ulna shows extensive periosteal reactive bone tissue with loss or discontinuity of the cortex in what appears to be multiple regions. It is therefore apparent that these features in the mamenchisaurid ulna are inconsistent with an osteoblastoma. Thus, it is unfortunate that although we can deduce that the pathology is an osteogenic tumor, a more specific diagnosis cannot be made with confidence.

### 4.2 Paleobiological implications of the osteogenic tumor in the ulna of the mamenchisaurid

**FIGURE 6:**
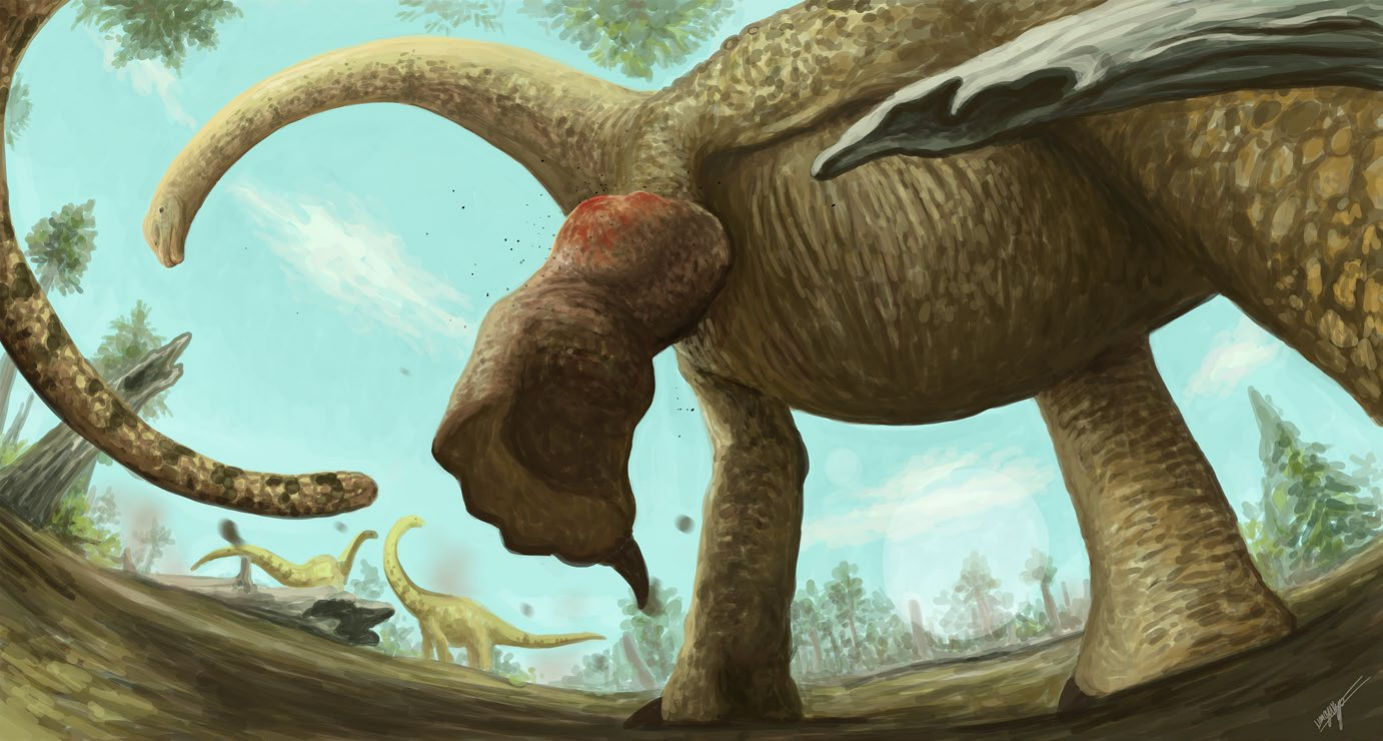
A reconstruction of a mamenchisaurid with a pathology evident in its left forelimb. Artwork by Kmonvich Lawan.

In order to analyze the potential effect of the osteogenic tumor on the mamenchisaurid's locomotion, we need to consider the muscles and tendons that are attached to the pathologic region as well as its neighboring areas (Cruzado‐Caballero et al., 2021). In the study that reported on the osteoblastoma in the vertebra of the hominin *Australopithecus sediba* from Malapa, South Africa, Randolph‐Quinney et al. (2016) suggested that since the lesion was close to inserting muscles such as the *trapezius*, *erector spinae*, and *rhomboid major*, it would have restricted the movement of both the shoulder blade and the upper right quadrant of the back. Thus, normal movement would have been impacted, especially considering the likely arboreal lifestyle of *A. sediba*. In the Thai mamenchisaurid, the extent and severity of the osteogenic tumor, as well as its location to musculoskeletal muscles such as the *flexor digitorum longus*, which is important for digit flexion, and *triceps brachii*, which controls movements of the elbow (Otero et al., 2017), it is highly likely that the lesion may have affected normal musculoskeletal function and movement of its forelimb (Figure 6), which probably caused severe discomfort, especially considering that bone‐forming tumors are known to involve physiological responses such as pain and muscular spasms.

### 4.3 Ontogenetic status

Based on histological features evident in the compacta of sauropodomorph dinosaurs, and especially the degree of secondary reconstruction, Klein and Sander (2008) proposed different histological ontogenetic stages (HOS) which can be used to determine the ontogenetic stages of sauropods. Considering that our mamenchisaurid ulna has intensive vascularization, large primary osteons, abundant secondary osteons, and at least three growth marks in the outer cortex, it appears to correlate with HOS 7 (Klein & Sander, 2008; Sander et al., 2011), which suggests that it had passed its most rapid phase of growth, but was still a growing individual.

### 4.4 Taphonomic fractures

The sagittal image showed evidence of a breakage of the ulna on the posterior side of the metaphysis (Figures 1c and 3). However, there is no evidence of deformation or associated callus formation, and the fractures are infilled with mud or sediment (Kato et al., 2020; Peterson & Vittore, 2012). These features suggest that all the fractures are taphonomic and were associated with postmortem crushing or burial and therefore exclude any post‐traumatic processes.

## 5 CONCLUSIONS

The paleopathology in the ulna of the 150‐million‐year‐old mamenchisaurid from Thailand is identified as having an osteogenic tumor in the posterior metaphysis of the bone. Using a combination of anatomical observations, CT scan data, and osteohistology of the afflicted region of the ulna, we can rule out several pathologies (such as osteosarcoma, osteomyelitis, intraosseous abscess, stress fracture, and hemangioma), but the nature of the specific pathology in the mamenchisaurid remains elusive. Definitive diagnoses of a bone pathology in modern animals can sometimes be extremely challenging (e.g., Maxie, 2015), especially because different bone tumors often have similar characteristics (e.g., Thompson & Dittmer, 2017). Thus, here we prefer to err on the side of caution, fully aware that we do not even have soft tissue to assist in the diagnosis. The osteohistological data permitted the identification of the individual as a subadult, that is, an individual that had passed its most rapid phase of growth but was still growing. In addition, using both the CT scan data and the histological sections, we were able to identify the fractures in the bone as unrelated to the pathologic response and likely caused by taphonomic damage to the bone. Overall, our findings contribute to knowledge of dinosaur paleopathologies and shed insight into the biology of basal sauropods.

## Supporting information


Table S1.

## Data Availability

Our histology images will be uploaded onto morphobank.
