# Supplementary material for: Novel report of an osteogenic tumor in a late Jurassic Mamenchisaurid from Thailand
Source: J Anat. 2025 Apr 24;247(3-4):699–711. doi: 10.1111/joa.14266 (PMC12397083; doi:10.1111/joa.14266)
Supplement: Supplementary file 1 — Table S1. [file JOA-247-699-s001.docx]

Supplementary Table 1: Extended differential diagnosis for specimen PN14-108

| Pathology | Diagnosis | Characteristics | | | | | | | | |
| --- | --- | --- | --- | --- | --- | --- | --- | --- | --- | --- |
|  |  | **Location** | **Shape** | **Border** | **Edge** | **Density** | **Cortex** | **Size** | **Periosteal reaction** | **Sequestra** |
| This study | PN14-108 | C | Irregular | W | S | Mixed lytic and sclerotic | Breakthrough | L | Spicule | Absent |
| Infection | Abscess | M or C | Elliptical | W/ I | N/ S | Lytic | Breakthrough | V | Absent | Present |
|  | Osteomyelitis | M or C | Irregular | W/ I | N | Lytic/ Sclerotic | Breakthrough | V | Spiculate/  Codman triangle | Present |
|  | Fungal infection |  | Spherical | W | N | Lytic | Breakthrough | V | Absent | - |
| Benign bone tumor | Osteoid osteoma | C | Spherical/  Elliptical | W | N | Nidus | Normal | Sm | Solid | Absent |
|  | Osteoblastoma | M or C | Sphere | W | N/ S | Lytic/ Sclerotic | Normal/ Break-through (aggressive) | L | Solid | Absent |
|  | Giant cell tumor | Eccentric | Irregular | W/ I | N | Lytic | Breakthrough | V | Absent | - |
|  | Chondromyxoid fibroma | M | Elliptical | W | S | Lytic | Thinned/  thickened | - | Absent | - |
|  | Hemangioma | M to eccentric | Linear/  elliptical | W/ I | N | Lytic | Breakthrough | - | Absent | - |
|  | Chondroblastoma | M of Epiphysis | Lobulated | W | S | Lytic | Scalloped | - | Present | - |
| Malignant bone tumor | Intracortical osteosarcoma | C | long ovoid | W | N | Lytic | Normal | Sm | Present | Absent |
| Non  neoplasm | Simple bone cyst | M | Elliptical | W | N | Lytic | Normal | - | Absent | - |
|  | Non-ossifying fibroma | M | Irregular | W | S | Lytic | Normal | - | Absent | - |
|  | Aneurysmal bone cyst | M to eccentric | Soap  bubble | W | S | Lytic | Normal | V | Absent | Absent |
|  | Sarcoidosis | - | Elliptical | W | N/ S | Lytic | Normal | - | Absent | - |
|  | Amyloidosis | - | Spherical | W | N | Lytic | Normal | - | Absent | - |
|  | Gout | Epiphysis | Spherical | W | N/ S | Lytic | Normal | - | Absent | - |

Explanations: Location: M, medullary cavity; C, intracortical; Border: W, well-defined; I, ill-defined; Edge: N, normal; S, sclerotic; Size: L, large; Sm, small; V, variable; Cortex terminology refers to the extent of disruption of the cortex.
